# Prevalence of *Listeria monocytogenes* in milk in Africa: a generalized logistic mixed-effects and meta-regression modelling

**DOI:** 10.1038/s41598-023-39955-0

**Published:** 2023-08-04

**Authors:** Yinka D. Oluwafemi, Bright E. Igere, Temitope C. Ekundayo, Oluwatosin A. Ijabadeniyi

**Affiliations:** 1 Department of Microbiology, University of Medical Sciences, Ondo, Nigeria; 2 Department of Microbiology, Dennis Osadebay University Anwai, Asaba, Delta State Nigeria; 3 Department of Biotechnology and Food Science, Durban University of Technology, Steve Biko Campus, Steve Biko Rd, Musgrave, Berea, Durban, 4001 South Africa

**Keywords:** Applied microbiology, Policy and public health in microbiology, Microbiology, Food microbiology

## Abstract

*Listeria* outbreaks and food recalls is on the raise globally. Milk particularly is highly susceptible to *Listeria* as its production and storage adequately support *Listeria* growth. The extent of milk contamination with *Listeria monocytogenes* (*Lm*) and preventative actions to halt milk associated outbreaks in Africa are unknown. Hence, this study aimed at assessing the national and subregional prevalence of *Lm* in milk in Africa and identify impacting factors via generalized logistic mixed-effects (GLMEs) and meta-regression modelling. *Lm*-milk-specific data acquired from primary studies according to standard protocol were fitted using a GLMEs. The GLMEs was subjected to leave-one-study-out-cross-validation (LOSOCV). Factors impacting *Lm* prevalence in milk were assayed via a 1000-permutation-assisted meta-regression-modelling. The pooled prevalence of *Lm* in milk in Africa was 4.35% [2.73–6.86] with a prediction interval (PI) of 0.14–59.86% and LOSOCV value of 2.43% [1.62–3.62; PI: 0.32–16.11%]. Western Africa had the highest prevalence [20.13%, 4.13–59.59], then Southern Africa [5.85%, 0.12–75.72], Northern Africa [4.67%, 2.82–7.64], Eastern Africa [1.91%, 0.64–5.55], and there was no record from Central Africa. In term of country, *Lm* prevalence in milk significantly (p < 0.01) varied from 0.00 to 90.00%. Whereas the *Lm* prevalence was negligibly different (p = 0.77) by milk type, raw-milk had the highest prevalence [5.26%], followed by fermented-milk [4.76%], boiled-milk [2.90%], pasteurized-milk [1.64%], and powdered-milk [1.58%]. DNA extraction approach did not significantly (p = 0.07) affect *Lm* prevalence (Boiling [7.82%] versus Kit [7.24%]) as well as *Lm* detection method (p = 0.10; (ACP [3.64%] vs. CP [8.92%] vs. CS [2.27%] vs. CSP [6.82%]). Though a bivariate/multivariate combination of all tested variables in meta-regression explained 19.68–68.75% (R^2^) variance in *Lm* prevalence in milk, N, nation, and subregion singly/robustly accounted for 17.61% (F_1;65_ = 7.5994; p = 0.005), 63.89% (F_14;52_ = 4.2028; p = 0.001), and 16.54% (F_3;63_ = 3.4743; p = 0.026), respectively. In conclusion, it is recommended that adequate sample size should be prioritized in monitoring *Lm* in milk to prevent spuriously high or low prevalence to ensure robust, plausible, and credible estimate. Also, national efforts/interests and commitments to *Lm* monitoring should be awaken.

## Introduction

Microbial safety of milk (either raw or powdered milk) has received more interest in the recent times as many outbreaks have been linked with consumption of milk. Milk is composed of essential nutrients for the growth of microorganisms and several studies have revealed microbiological contamination and abundance and/or unsafe quality level at a high prevalence with the major culprits including *Listeria monocytogenes*^1,2^. Milk, a primary animal-based protein source in consumer’s diet occurs in varieties such as raw, fermented, powdered, and/or pasteurized milks^3,4^ with different degree of microbial exposure and contaminations. Following the dietary relevance of milk, composition and its associated preservation strategy, milk has become the major module for bacterial proliferation and contamination^5^. Various groups of bacterial as well as fungal pathogens have been reported to harness variety of human employed strategies involved in preparation/production, handling, storage, and production facilities of milk at specific points to perpetrate their survival and growth^5^. However, *L. monocytogenes* is of particular interest because of fatal outcomes of its infections especially in children, pregnant women, and immune-compromised individuals.

*L. monocytogenes,* a Gram-positive intracellular organism remains a chief contaminant in milk due to its ability to proliferate at low temperatures (refrigeration), water activity, pH, and high salinity. It is important to note that these conditions are notable man-made features provided to enhance prolong shelf-life, quality, and safety of milk before consumption^6,7^. Managing microbial contamination of milks include appropriate production sanitation and hygiene, training of handlers/farmers on good production practices including hygiene, use of portable low temperature equipment at cooperatives, improved milking methods, adequate improvement of production and transportation infrastructure, among others^8,9^. However, the contamination of milk by *L. monocytogenes* strains is almost unavoidable due to association of the bacteria with livestock breeding and management, their ability to form biofilm on production facilities, and high thermal resistance^10^. Overall, *L. monocytogenes* contamination without doubt affects the quality of milk and may also result in foodborne illness/outbreaks^11^. Such downturn and unwarranted consequences necessitated continuous surveillances and monitoring of milk safety to prevent economic loss and ensure public and consumer’s health.

With the increase in *Listeria* outbreaks and *Listeria*-associated food recalls, the state of *L. monocytogenes* contaminations in Africa especially in milks require attention. Milk been an important food in Africa and, one of the *Listeria* highly susceptible products due to its production and storage that adequately support microbial growth to a greater extent, command a more deliberate biosafety assessment. While global efforts to forestall its contamination and outbreaks is increasingly been advocated, it is unknown to what level such endeavours are being practice in Africa. Hence, this study aimed at assessing the national and subregional prevalence of *L. monocytogenes* in milk in Africa and identify various impacting factors via generalized logistic mixed-effects (GLMEs) and meta-regression modelling. To the best of our knowledge, this is the first GLMEs and meta-regression-based study on the subject in Africa and globally.

## Materials and methods

### Search strategy

Published studies in Africa on milk contamination by *Listeria monocytogenes* were strategically retrieved from PubMed, Scopus, and Web of Science (WoS) using the algorithm ‘*monocytogenes* AND milk*’ with refinement to African countries (database-specific details are presented supplementary material). The “Preferred Reporting Items for Systematic Reviews and Meta-analyses (PRISMA) guidelines”^12^ was employed for the search using topic-specific field on 6 January 2023 at 22.00 GMT.

### Inclusion and exclusion criteria

The present study considered primary studies that evaluated *Listeria monocytogenes* contamination in milks in Africa. First, the included study must be affiliated with an African nation, number of *L. monocytogenes* positive samples, sample size collected, *Lm* isolation method, *Lm* confirmation strategy (PCR, serology, cultural method, including DNA extraction method). Studies that lack specified relevant details above are excluded. Laboratory spike experimental studies, opinion documents, editorials and reviews were excluded from the study.

### Data management and extraction

The studies metadata on *Lm* contamination of milk acquired from different databases by one investigator (TE) was combined in Endnote version 20 and de-duplicated in Excel version 2016. Afterward, TE screened the unique studies' titles and abstracts for consideration. Then, the full-text of the eligible studies was retrieved, read, and data collated into predesigned Excel forms. The reference lists of the studies were further read for extra record(s). The entire workflow is represented in Fig. S1.

The data collated in 2 sets (OYD and IBE) from the studies were authors' name, positive sample size (P), publication year (PY), sample size (N), type of milk, *Lm* confirmation method (cultural/culture independent), DNA extraction procedure, nation, and subregion as derivatives of nations. The data extraction and quality assessments were done by OYD and IBE and designated as respective sets. The datasets were validated for equality as ∣OYD ∩ IBE∣ ≡ ∣OYD ∪ IBE∣ and where there was any variance, TE led discussion to resolve the differences.

### Statistical analysis

The final datasets comprising 6893 milk samples were subjected to explanatory analysis and subsequently standardized and fitted in GLMEs according to Eqs. (1) and (2) with a 0.5 continuity adjustment^13^.

1 $${\theta }_{s}^{\iota o}= {\mathrm{log}}_{e}\frac{{\rho }_{s}}{\left(1-{\rho }_{s}\right)} , {\theta }_{s}^{\iota o}\sim \theta + {u}_{s}, with\, {u}_{s} \sim N\left(0,{\tau }^{2}\right).$$

2 $${y}_{i}={\beta }_{0}+{\beta }_{1}{x}_{v}+{u}_{v}+{\epsilon }_{v}$$

where *p* = proportion (i.e., P/sample size), β_0_ = overall effect size (*Lm* prevalence), β_1_*x* = regression term, ${u}_{v}$ = random-effect term with ${u}_{v} \sim N\left(0,{\tau }^{2}\right)$, ${\in }_{v}=$ random error with ${\in }_{v}\sim N\left(0,{\Sigma }_{v}\right)$, β weights = the common effects elements.

In the GLMEs, the number of events in a study (${u}_{s}$) is presupposed to be distributed as:

$${u}_{s} \sim B\left({n}_{s}, \frac{\mathrm{exp}({t}_{s}^{\iota o})}{1+\mathrm{exp}({t}_{s}^{\iota o})}\right)$$

Higgins and Thompson (2002) method was applied in calculating I^2^ and H^2^ statistics (between-study heterogeneity) in the GLMEs (Eqs. 3 and 4).

3 $${I}^{2}=Q-(S-1)/Q$$

4 $${H}^{2}=Q/S-1$$

where $Q=\sum_{s=1}^{S}{\omega }_{s}{({\widehat{t}}_{s}-\widehat{t})}^{2}$ and ${\widehat{t}}_{s}-\widehat{t}\sim N\left(\mathrm{0,1}\right)$, mean $\widehat{t}$= overall effect according to the common-effect model; ${\omega }_{s}=$ weighting term; Where there is no heterogeneity, *Q* was assumed to follow a ${\chi }^{2}$ distribution with S − 1 degrees of freedom.

An I^2^ statistic ≥ 75% implied a remarkable degree of heterogeneity (Higgins and Thompson, 2002).

The robustness of the models in addition was demonstrated via leave-one-study-cross-validation, LOSOCV^14^ and Egger’s regression^15^. The study further explored sub-group generalized logistic-mixed-effects models (SgGLMEs) in assessing various group-specific prevalence and subgroup-specific differences^16^. Factors impacting *Lm* prevalence in milk were also assayed via a 1000-permutation-assisted meta-regression-modelling^17,18^ in which N and PY were inputted as continuous variables and milk type, detection method, country, and subregion as categorical elements.

### Software

The fitting of all equations/models including GLMEs, LOSOCV, SgGLMEs, 1000-permutation-assisted meta-regression-models and estimation of I^2^- and H^2^-statistics in R v.4.2.2 (2022-10-31 ucrt) were based on functions accrued from metafor v.3.8-1, meta v.6.1-0, PerformanceAnalytics v.2.0.4, and dmetar v.0.0.9000^19–22^.

## Results

**Figure 1:**
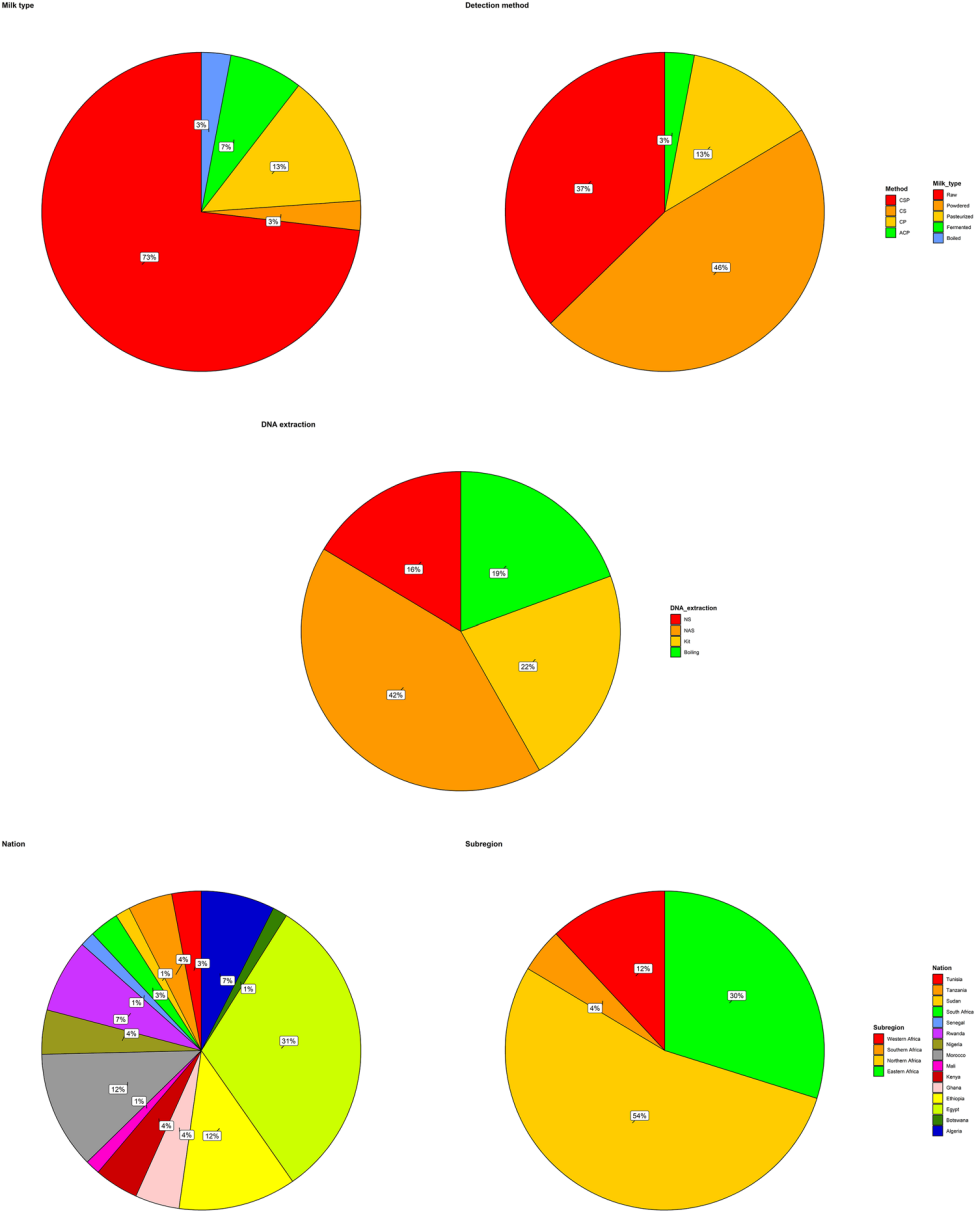
Descriptive summary of the included studies on *L. monocytogenes* contamination of milk in Africa. *ACP* API kit, cultural and PCR; *CP* cultural and PCR; *CS* cultural and serology; *CSP* cultural, serology, and PCR.

A descriptive summary of the included studies is presented in Fig. 1 and details Tables S1 and S2. A total of 67 disaggregated studies (N) with overall mean of 8.48 ± 18.28 and 102.88 ± 113.12 *L. monocytogenes* positive samples and sample size respectively, were acquired. The samples contained 73% raw milk, 13% pasteurized milk, 7.5% fermented milk, 3.0% boiled milk, and 3.0% powdered milk. The *L. monocytogenes* detection method employed in the studies include CS (cultural and serology; 31/67, 46.0%), CSP (cultural, serology, and PCR; 25/67, 37.0%), CP (cultural and PCR; 9/67, 13%), and ACP (API kit, cultural and PCR; 2/67, 3.0%). DNA extraction method was through boiling (13/67, 19.0%) and Kit (15/67, 22.0%) but not applicable in 28/67 (42%) or unspecified in 11/67 (16%) studies. Highest number of the studies were from Egypt (31%), followed by Ethiopia (12%) and Morocco (12%). Subregional characteristics showed that Northern Africa (54%) had highest studies, followed by the Eastern Africa (30%), Western Africa (12%), and Southern Africa (4.5%).

**Figure 2:**
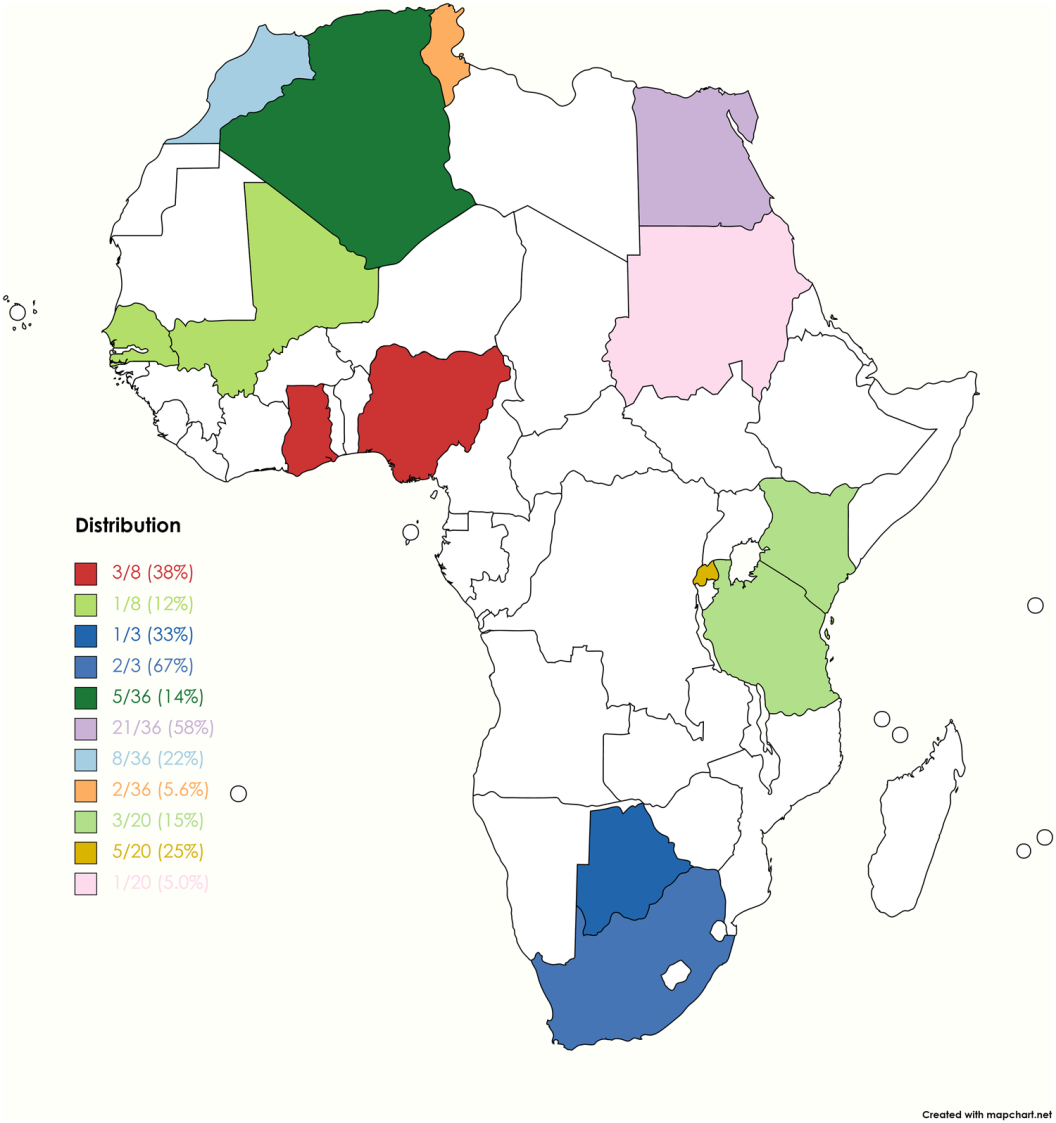
Subregional specific distribution of studies on *L. monocytogenes* contamination of milk in Africa.

The subregional specific distribution of studies on *L. monocytogenes* contamination of milk in Africa is showed in Fig. 2 and Table S3. Amongst subregions, Northern Africa showed the highest distribution (N = 36) with an average positive sample and sample sizes of 7.86 ± 14.65 and 88.11 ± 62.81 respectively. Eastern Africa had 20 with average *L. monocytogenes* positive sample and sample size of 4.25 ± 5.80 and 128.70 ± 173.88, Western Africa had 8 with average *L. monocytogenes* positive sample and sample size of 23.50 ± 40.81 and 99.50 ± 92.46 respectively, and Southern Africa had 3 studies with average *L. monocytogenes* positive sample and sample size of 4.00 ± 2.65 and 117.00 ± 158.48 respectively.

### Overall and subgroup pooled prevalence of *L. monocytogenes* contamination in African milk

The overall and subgroup pooled prevalence of *L. monocytogenes* contamination in milk in Africa is summarized in Table 1. The pooled prevalence of *L. monocytogenes* in milk in Africa was 4.35% [2.73–6.86; I^2^ = 89.7%, 87.6–91.4, p < 0.0001] with a prediction interval (PI) of 0.14–59.86% and LOSOCV value of 2.43% [1.62–3.62; PI: 0.32–16.11%; I^2^ = 39.0%, 13.4–57.0, p = 0.0037]. Western Africa had the highest prevalence [20.13%, 4.13–59.59; I^2^ = 97%, 96–98, p < 0.01], then Southern Africa [5.85%, 0.12–75.72; I^2^ = 92%, 79–97, p < 0.01], Northern Africa [4.67%, 2.82–7.64; I^2^ = 82%, 75–86, p < 0.01], Eastern Africa [1.91%, 0.64–5.55; I^2^ = 81%, 72–88, p < 0.01], and no record from Central Africa.

**Table 1:** Overall and group pooled prevalence of *L. monocytogenes* contamination in African milk.

	Prevalence % [95% CI]	PI	I^2^% [95% CI]	Q [df; p-value]
Overall pooled prevalence	4.35 [2.73–6.86]	[0.14–59.86]	89.7 [87.6–91.4]	637.74 [66; p < 0.0001]
LOSOCV	2.43 [1.62–3.62]	[0.32–16.11]	39.0 [13.4–57.0]	77.02 [47; p = 0.0037]
DNA extraction approach				
Boiling	7.82 [4.00–14.73]		86 [78–91]	85.36 [12; p < 0.01]
Kit	7.24 [2.44–19.60]		94 [92–96]	238.18 [14 (p < 0.01]
Not applicable	2.03 [0.83–4.85]		75 [63–82]	106.09 [28; p < 0.01]
Not specified	5.24 [1.31–18.74]		86 [77–92]	72.32 [10; p < 0.01]
Test for DNA extraction differences p = 0.07				
*L. monocytogenes* detection method				
ACP	3.64 [0.01–96.06]		0	0.03 [1; p = 0.85]
CP	8.92 [1.21–43.80]		70 [41–85]	27.02 [8; p < 0.01]
CS	2.27 [1.03–4.90]		78 [70–85]	138.79 [30; p < 0.01]
CSP	6.82 [3.72–12.18]			387.54 [24; p < 0.01]
Test for *L. monocytogenes* detection differences: p = 0.10				
Subregion				
Eastern Africa	1.91 [0.64–5.55]		81 [72–88]	102.14 [19; p < 0.01]
Northern Africa	4.67 [2.82–7.64]		82 [75–86]	189.95 [35; p < 0.01]
Southern Africa	5.85 [0.12–75.72]		92 [79–97],	24.33 [2; p < 0.01]
Western Africa	20.13 [4.13–59.59]		97 [96–98]	231.36 [7; p < 0.01]
Test for subregional differences: p = 0.05				
Nation				
Algeria	1.27 [0.15–10.27]		24 [0–69]	5.29 [4; p = 0.26]
Botswana	1.00 [0.21–2.89]		NA	NA
Egypt	6.23 [3.33–11.34]		85 [79–90]	135.14 [20; p < 0.01]
Ethiopia	5.28 [2.05–12.93]		89 [82–94]	66.28 [7; p < 0.01]
Ghana	12.32 [6.07–23.41]		19 [0–92]	2.48 [2; p = 0.29]
Kenya	11.61 [1.82–48.15]		66 [0–90]	5.91 [2(p = 0.05]
Mali	90.00 [55.50–99.75]		NA	NA
Morocco	3.71 [1.22–10.72]		55 [0–80]	15.52, df-7; p = 0.03]
Nigeria	5.56 [0.13–73.47]		89 [71–96]	18.65 [2; p < 0.01]
Rwanda	0.00 [0.00–100.00]		0 [0–79]	0 [4; p = 1.00]
Senegal	81.58 [74.49–87.40]		NA	NA
South Africa	16.76 [0.03–99.17]		65 [0–92]	2.82 [1; p = 0.09]
Sudan	0.42 [0.09–1.21]		NA	NA
Tanzania	0.00 [0.00–100.00]		0 [0–90]	0 [2; p = 1.00]
Tunisia	16.44 [0.35–91.58]		0	0.8 [1; p = 0.37]
Test for national differences: p < 0.01				
Milk type				
Boiled	2.90 [0.00, 100.00]		0	0 [1; p = 1.00]
Fermented	4.76 [0.53–31.98]		0 [0–79]	0.97, p = 0.91
Pasteurized	1.64 [0.14–16.12]		0 [0–65]	5.87, p = 0.66
Powdered	1.58 [0.00–100.00]		0	0 [1; p = 1.00]
Raw	5.26 [3.00–7.39]		95 [91, 94]	618.02 [48; p < 0.01]
Test for milk type differences: p = 0.77				

The *L. monocytogenes* prevalence in milk was significantly different (p < 0.01) across countries and valued from 0.00 to 90.00%. Mali had the highest *L. monocytogenes* prevalence in milk (90.00%, 55.50–99.75), followed by Senegal [81.58%, 74.49–87.40], South Africa [16.76%, 0.03–99.17; I^2^ = 65%, 0–92, p = 0.09], Tunisia [16.44%, 0.35–91.58; I^2^ = 0, p = 0.37], Ghana [12.32%, 6.07–23.41; I^2^ = 19%, 0–92, p = 0.29], Kenya [11.61%, 1.82–48.15; I^2^ = 66%, 0–90, = 0.05], Egypt [6.23%, 3.33–11.34; I^2^ = 85%, 79–90, p < 0.01], Nigeria [5.56%, 0.13–73.47; I^2^ = 89%, 71–96, p < 0.01], Ethiopia [5.28%, 2.05–12.93; I^2^ = 89%, 82–94, p < 0.01], Morocco [3.71%, 1.22–10.72; I^2^ = 55%, 0–80, p = 0.03], Algeria [1.27%, 0.15–10.27; I^2^ = 24%, 0–69, p = 0.26], Botswana [1.00%, 0.21–2.89], Sudan [0.42%, 0.09–1.21], Rwanda [0.00%, 0.00–100.00; I^2^ = 0%, 0–79, p = 1.00], and Tanzania [0.00%, 0.00–100.00; I^2^ = 0%, 0–90, p = 1.00].

Whereas the *L. monocytogenes* prevalence was negligibly difference (p = 0.77) by milk type, raw-milk had the highest prevalence [5.26%, 3.00–7.39; I^2^ = 95%, 91–94, p < 0.01], followed by fermented-milk [4.76%, 0.53–31.98; I^2^ = 0%, 0–79, p = 0.91], boiled-milk [2.90%, 0.00–100.00, I^2^ = 0, p = 1.00], pasteurized-milk [1.64%, 0.14–16.12; I^2^ = 0%, 0–65, p = 0.66], and powdered-milk [1.58%, 0.00–100.00; I^2^ = 0, p = 1.00]. DNA extraction approach did not significantly (p = 0.07) affect *L. monocytogenes* prevalence (Boiling [7.82%, 4.00–14.73; I^2^ = 86%, 78–91, p < 0.01] versus Kit [7.24%, 2.44–19.60; I^2^ = 94%, 92–96, p < 0.01). Similarly, *L. monocytogenes* detection method did not significantly influence *L. monocytogenes* prevalence (p = 0.10) in milk but was highest by CP [8.92%, 1.21–43.80; I^2^ = 70%, 41–85; p < 0.01], followed by CSP [6.82%, 3.72–12.18; p < 0.01], ACP [3.64%, 0.01–96.06; I^2^ = 0, p = 0.85] and CS [2.27%, 1.03–4.90; I^2^ = 78%, 70–85, p < 0.01].

### Meta-regression identification of the various factors and moderating influences contributing to the prevalence of* L. monocytogenes* contamination in African milks

Table 2 shows the univariate, bivariate and multivariate meta-regression of the various factors/moderating influences contributing to the prevalence of *L. monocytogenes* contamination in African milks. Meanwhile a bivariate/multivariate combination of all tested variables in meta-regression explained 19.68–68.75% (R^2^) variance in *L. monocytogenes* prevalence in milk with some combination significant regression weights, N, nation, and subregion singly/robustly accounted for 17.61% (F_1;65_ = 7.5994; p = 0.005), 63.89% (F_14;52_ = 4.2028; p = 0.001), and 16.54% (F_3;63_ = 3.4743; p = 0.026), respectively. Specifically, a bivariate addition of N & Method, N & Subregion, N & DNA extraction, and N & Nation had a significant regression weight of − 3.0571 ± 1.0095 (p = 0.01), − 2.4827 ± 0.3932 (p = 0.001), − 1.9577 ± 0.4002 (p = 0.001), and − 3.2446 ± 0.5864 (p = 0.001), respectively, and explained 28.61% (F_4;62_ = 3.5414; p = 0.015), 31.94% (F_4;62_ = 4.7847; p = 0.002), 25.95% (F_4;62_ = 3.2114; p = 0.018) and 70.55% (F_15;51_ = 4.6072; p = 0.001) variance with a robust moderator test presented in parentheses in *L. monocytogenes* prevalence in milk in Africa respectively. In addition, it was generally observed that any bivariate/multivariate model containing at least N or nation resulted into a significant and robust moderator test.

**Table 2:** Meta-regression identification of the various factors and moderating influences contributing to the prevalence of *L. monocytogenes* contamination in African milks.

Univariate/bivariate/multivariate	β_0_ ± SE	τ^2^	I^2^ (%)	H^2^ (%)	R^2^ (%)	Test of Moderators (F_d1;d2_ = Q; p)
Nation, subregion & method	− 4.4482 ± 1.0825***	0.5439 ± 0.1493	72.54	3.64	68.75%	F_17;49_ = 3.5961; p = 0.003
Subregion & method	− 3.3260 ± 1.0594**	1.3577 ± 0.3095	87.23	7.83	21.99%	F_6;60_ = 2.1436; p = 0.061
PY, subregion & method	12.2000 ± 54.3912	1.3588 ± 0.3097	87.19	7.8	21.93%	F_7;59_ = 1.8209; p = 0.102
N, subregion & method	− 2.9687 ± 1.0099**	1.0746 ± 0.2551	84.04	6.27	38.26%	F_7;59_ = 3.2012; p = 0.009
Subregion, method & DNA extraction	− 3.3295 ± 1.2612*	1.3252 ± 0.3033	86.43	737.00	23.86%	F_9;57_ = 1.4248; p = 0.187
N, milk type & subregion	− 3.3424 ± 1.1219**	1.1249 ± 0.2649	84.98	666.00	35.37%	F_8;58_ = 2.6243; p = 0.026
N, subregion, method, nation & DNA extraction	− 5.8227 ± 1.8951**	0.3828 ± 0.1153	61.09	257.00	78.01%	F_25;41_ = 2.8228; p = 0.005
Milk type	− 2.4881 ± 1.1651*	1.7218 ± 0.3780	90.47	10.50	1.08	F_4;62_ = 0.2317; p = 0.925
Subregion	− 3.1398 ± 0.3411***	1.4526 ± 0.3275	88.73	8.87	16.54	F_3;63_ = 3.4743; p = 0.026
Method	− 3.2728 ± 1.0547**	1.5125 ± 0.3388	88.75	8.89	13.10	F_3;63_ = 2.1524; p = 0.092
Nation	− 3.7204 ± 0.5761	0.6286 ± 0.1667	77.21	4.39	63.89	F_14;52_ = 4.2028; p = 0.001
N	− 2.1548 ± 0.2466***	1.4340 ± 0.3240	88.71	8.85	17.61	F_1;65_ = 7.5994; p = 0.005
DNA extraction	− 2.3873 ± 0.3945***	1.5620 ± 0.3481	89.06	9.14	10.25	F_3;63_ = 1.5834; p = 0.194
N & Milk type	− 2.1857 ± 1.1012	1.3979 ± 0.3171	88.18	8.46	19.68	F_5;61_ = 1.8521; p = 0.115
N & Method	− 3.0571 ± 1.0095**	1.2425 ± 0.2875	86.36	733.00	28.61	F_4;62_ = 3.5414; p = 0.015
N & Subregion	− 2.4827 ± 0.3932***	1.1845 ± 0.2764	86.08	7.19	31.94	F_4;62_ = 4.7847; p = 0.002
N & DNA extraction	− 1.9577 ± 0.4002***	1.2888 ± 0.2964	86.66	750.00	25.95	F_4;62_ = 3.2114; p = 0.018
N & Nation	− 3.2446 ± 0.5864***	0.5126 ± 0.1428	72.28	361.00	70.55	F_15;51_ = 4.6072; p = 0.001

‘***’ 0.001 ‘**’ 0.01 ‘*’ 0.05 '^.^' 0.1.

## Discussion

The current study describes national and subregional prevalence of *L. monocytogenes* in milk in Africa. The overall mean sample positivity of *Lm* and the average sample size was 8.48 ± 18.28 and 102.88 ± 113.12, respectively. Thus, ≈ 103 sample size might be inadequate for monitoring *Lm* in milk as it would only be sensitive to yield ≈ 9 *Lm* positive samples. Sample size inadequacy could contribute to failed surveillance of *Lm* in milk. For instance, N explained 17.61% variance in *Lm* prevalence (Table S2), denoting a huge portion or contribution of sample size to adequate prevalence/surveillance of *Lm* in milk. A previous study found that difference in *Listeria* spp. isolation rate is in part influenced by sample size and isolation methods^23^. The various range of milk sample size in literature for monitoring *Lm* contamination ranged from 4 to 720 (Table S2) and disadvantageously distributed. For instance, the skewness of P and N in this study also indicated a substantial greater number of low *Lm* positivity and smaller sample size, respectively. Similarly, the kurtosis of P and N were more than + 2 indicating a distribution more peaked than normal. Generally, a skewness value between − 1 and + 1, − 2 and + 2, and beyond − 2 and + 2 is respectively considered as excellent, acceptable, and substantial nonnormality^24^. A positive value for the kurtosis indicates a distribution more peaked than normal. Correspondent to the skewness, a kurtosis >+ 2 and <− 2 is considered a distribution that is too peaked or too flat respectively^24^.

The variety of milk assayed for *Lm* ranged from 73% raw milk, 13% pasteurized milk, 7.5% fermented milk to 3.0% boiled milk and 3.0% powdered milk. This is an indication that more surveillance of *Lm* in pasteurized, fermented, boiled, and powdered milks should be intensified in addition to raw milk. *Lm* is known to possess thermal resistance and withstand desiccation. The *Lm* detection method included CS (46.0%), CSP (37.0%), CP (13%), and ACP (3.0%). Although no method showed superiority over another in the detection of *Lm* in milk (Table 1), PCR-based methods have higher likelihoods to eliminate false-positive/misdiagnosis in detecting Lm compared with other methods. The application of kit (22.0%) in DNA extraction method was found to be higher than boiling (19.0%). However, both methods had equal performance in relation to *Lm* detection in milk (Table 1). More attention to the monitoring of *Lm* in milk was found in Northern Africa, followed by the Eastern Africa, Western Africa, and Southern Africa.

This connotes differences in subregional Lm monitoring strategy in milk and require a general step up across the geographical locations.

The findings of the present study indicated a pooled prevalence of *Lm* in milk in Africa as 4.35% coupled with a higher prediction limit of 59.86%. thus, suggest possible general underestimation of *Lm* in Africa due to inactions and inadequate monitoring programs in the various subregions. Meanwhile, *Lm* prevalence was highest in the Western Africa [20.13%], followed by Southern Africa [5.85%], Northern Africa [4.67%], Eastern Africa [1.91%], and no record from Central Africa. On nation-basis, Mali had highest prevalence (90.00), followed by Senegal [81.58%], South Africa [16.76%], Tunisia [16.44%], Ghana [12.32%], Kenya [11.61%], Egypt [6.23%], Nigeria [5.56%], Ethiopia [5.28%], Morocco [3.71%], Algeria [1.27%], Botswana [1.00%], Sudan^4^, [0.42%], Rwanda [0.00%], and Tanzania [0.00%].

Individual studies from Western Africa have reported varied prevalence of *Lm* from countries in the subregion including 0.00%, 3.82%^25^, and 25.00%^26^ in raw milk in Nigeria in Nigeria; 9.72%^27^, 13.10%^27^, and 17.86%^27^ in raw, Nunu/Fermented, and Boiled milk respectively, in Ghana; 81.58%^28^ in raw in Senegal, and 90.00% in raw in Mali^29^. On the overall, high level of *Lm* contamination in milk appeared to be a major concern in the Western Africa and require a state of emergency. Also, individual studies from Southern Africa have reported *Lm* prevalence in milk as 1.00% in raw milk in Botswana^30^, 8.00% in pasteurized milk and 26.92% in raw milk in South Africa^1^. In the Northern Africa subregion, *Lm* prevalence from individual studies in milk ranged from 0.00 to 2.61% in raw and pasteurized milk in Algeria^31–34^, 0.0% in pasteurized milk (Ahmed et al. 2022), 0.0–5.63% in powdered milk^6,35^, and 0.00–34.00% in raw milk^8,11,36–51^; in Egypt. Previous individual studies on *Lm* contamination of milk in the Eastern African reported 0.00%^52^ and 20.00%^53^ in pasteurized milk, 2.34–22.00%^7,52,54–57^ in raw milks in Ethiopia; 21.43%^58^ in raw milk, 0.00% in boiled milk^59^, 0.00% in fermented milk^59,60^, and 0.00%^59^ in pasteurized milk in Kenya; 0.00%^59^ and 0.42%^61^ in raw milk in Rwanda and Sudan respectively, 0.00% in fermented, pasteurized, and raw milks in Tanzania^62^.

The observed negligibly difference of *L. monocytogenes* prevalence in the milk types, with raw-milk having the highest prevalence [5.26%], followed by fermented-milk [4.76%], boiled-milk [2.90%], pasteurized-milk [1.64%], and powdered-milk [1.58%], implies that all kinds of milk possessed *Lm* health risk to consumers and should adequately be monitored.

Whereas the previous studies found that differences in *Listeria* isolation methods impact the isolation rate^23^, the effects of different procedures involved in the confirmation of *Lm* has not been reported. Here, neither the use of kit nor boiling method in DNA extraction affects accurate estimate of *Lm* prevalence in milk. The advocacy or believed of the superiority of the use of kit over boiling method of DNA extraction in some quarters should be dispelled. Likewise, *Lm* detection method bear no significant influence on *Lm* prevalence in milk attesting to their capability to achieve accurate sensing of *Lm* in milk samples.

Furthermore, this study found sample size (N), nation, and subregion as robust factors that influence the incidence and prevalence of *Lm* in milk Africa and respectively, accounted for 17.61%, 63.89%, and 16.54% of the variance. Generally, sample size as two common effects on prevalence estimate as well as other effect size measures. An inadequate sample size would either yield false-negative outcome or produce a spuriously high or low prevalence estimate. On the other hands, adequate sample size will generate a robust prevalence estimate as drawing from a large pool of samples increase plausibility, confidence, and credibility of such estimate. The number of expected samples should be determined beforehand using an appropriate sample size determination formula based on prevalence of a pathogen (*Lm*) reported in infectious conditions or foodborne contaminations in previous studies with relatively large sample sizes. Specific bivariate addition of N and other factors such as method, subregion, DNA extraction approach, and nation possessed a significant regression weight respectively explained 28.61%, 31.94%, 25.95%and 70.55% variance in *L. monocytogenes* prevalence in milk in Africa. This further attests to the relevance of sample size in *Lm* accurate prevalence estimates and must be taken into consideration at the very beginning of the design of any study. *Listeria* spp. isolation rate is partly influenced by sample size and isolation methods^23^. The identification of nation as a key factor in prevalence of *Lm* in milk can be adduced to cultural differences in milk productions, differences in *Lm* monitoring commitments, and practices among countries and subregions among others. For instance, difference in MRSA prevalence in meats (among nations) has been attributed to differences in sample size variations, handling practices, geographical locations, experimental methods, seasonal variations, and management practices^63^.

## Conclusion

The current study foregrounds that *Lm* monitoring in milks in Africa was generally low and distributed as 73% raw milk, 13% pasteurized milk, 7.5% fermented milk, 3.0% boiled milk, and 3.0% powdered milk with an overall average sample size of ≈103. Higher surveillance of *Lm* in milk were seen from Egypt in contrast with other countries and in the subregion of Northern Africa compared with the Eastern Africa, Western Africa, Southern Africa and with no record from the Central Africa. While the pooled prevalence of *Lm* in milk in Africa was 4.35% with an upper prediction limit of 59.86% revealing potential underestimation, *Lm* had higher prevalence in milk above the pooled prevalence in Western Africa [20.13%], Southern Africa [5.85%], and Northern Africa [4.67%]. Specifically, *Lm* prevalence decreased from Mali (90.00), Senegal [81.58%], South Africa [16.76%], Tunisia [16.44%], Ghana [12.32%], Kenya [11.61%], Egypt [6.23%], Nigeria [5.56%], Ethiopia [5.28%], Morocco [3.71%], Algeria [1.27%], Botswana [1.00%], Sudan [0.42%] to Rwanda [0.00%], and Tanzania [0.00%]. In addition, negligibly difference of *Lm* prevalence in different kinds of milk, with highest prevalence in raw-milk [5.26%], followed by fermented-milk [4.76%], boiled-milk [2.90%], pasteurized-milk [1.64%], and powdered-milk [1.58%], implies equality in *Lm* health risk from the milk varieties. In addition, sample size (N), nation, and subregion were robust factors that influence the incidence and prevalence of *Lm* in milk Africa, accounting for at least 17.61%, 63.89%, and 16.54% variance, respectively. It is recommended that adequate sample size and homogeneous sampling strategy should be prioritized and determined ahead using an appropriate sample size determination formula in monitoring *Lm* in milk to prevent false-negative outcomes and spuriously high or low prevalence estimate to ensure robust, plausible, and credible estimate. Also, national efforts and commitments to *Lm* monitoring should be pursued and awaken as identification of nation/subregion as a fundamental factor in *Lm* prevalence in milk showed cultural differences in milk production, *Lm* monitoring interests, and practices among countries and subregions.

### Supplementary Information

Supplementary Information.

## Data Availability

All data generated or analysed during this study are included in this published article and its supplementary information file.
