# Supplementary material for: Prevalence of Listeria monocytogenes in milk in Africa: a generalized logistic mixed-effects and meta-regression modelling
Source: Sci Rep. 2023 Aug 4;13:12646. doi: 10.1038/s41598-023-39955-0 (PMC10403535; doi:10.1038/s41598-023-39955-0)
Supplement: Supplementary file 1 — Supplementary Information. [file 41598_2023_39955_MOESM1_ESM.docx]

**Supplemental materials for ‘National and subregional prevalence of *Listeria monocytogenes* in milk in Africa: a generalized logistic mixed-effects and meta-regression modelling’**

**Yinka D. Oluwafemi ^a^; Bright E. Igere^b^; Temitope C. Ekundayo^a,c*^; Oluwatosin A. Ijabadeniyi^c^**

^a^Department of Microbiology, University of Medical Sciences Ondo, Nigeria

^b^Department of Microbiology, Dennis Osadebay University Anwai, Asaba, Delta State, Nigeria.

^c^Department of Biotechnology and Food Science, Durban University of Technology, Steve Biko Campus, Steve Biko Rd, Musgrave, Berea, 4001, Durban, South Africa*.*

*corresponding author’s E-mail: cyruscyrusthem@gmail.com

**Methods**

**PubMed**
66 results

(Monocytogenes*[tiab] & milk*[tiab]) & (Nigeria[ad] | Ethiopia*[ad] | Egypt*[ad] | Congo*[ad] | Tanzania*[ad] | Africa*[ad] | Kenya*[ad] | Uganda*[ad] | Algeria*[ad] | Sudan*[ad] | Morocco*[ad] | Angola*[ad] | Mozambique*[ad] | Ghana*[ad] | Madagascar*[ad] | Cameroon*[ad] | Côte d'Ivoire *[ad] | Niger*[ad] | Burkina*[ad] | Mali*[ad] | Malawi*[ad] | Zambia*[ad] | Senegal*[ad] | Chad*[ad] | Somalia*[ad] | Zimbabwe*[ad] | Guinea*[ad] | Rwanda*[ad] | Benin*[ad] | Burundi*[ad] | Tunisia*[ad] | Togo*[ad] | Sierra*[ad] | Libya*[ad] | Congo*[ad] | Liberia*[ad] | Mauritania*[ad] | Eritrea*[ad] | Namibia*[ad] | Gambia*[ad] | Botswana*[ad] | Gabon*[ad] | Lesotho*[ad] | Guinea-Bissau*[ad] | Equatorial Guinea*[ad] | Mauritius*[ad] | Eswatini*[ad] | Djibouti*[ad] |Comoros*[ad] | Cabo Verde*[ad] | Principe*[ad] | Seychelles*[ad] | Réunion *[ad] | Western Sahara*[ad] |Mayotte*[ad] | Helena*[ad])

Scopus
[145](tel:145) document results

TITLE-ABS-KEY ( monocytogenes  AND  milk* )  AND  ( LIMIT-TO ( DOCTYPE ,  "ar" ) )  AND  ( LIMIT-TO ( AFFILCOUNTRY ,  "Egypt" )  OR  LIMIT-TO ( AFFILCOUNTRY ,  "Morocco" )  OR  LIMIT-TO ( AFFILCOUNTRY ,  "Tunisia" )  OR  LIMIT-TO ( AFFILCOUNTRY ,  "Algeria" )  OR  LIMIT-TO ( AFFILCOUNTRY ,  "Ethiopia" )  OR  LIMIT-TO ( AFFILCOUNTRY ,  "Nigeria" )  OR  LIMIT-TO ( AFFILCOUNTRY ,  "South Africa" )  OR  LIMIT-TO ( AFFILCOUNTRY ,  "Kenya" )  OR  LIMIT-TO ( AFFILCOUNTRY ,  "Ghana" )  OR  LIMIT-TO ( AFFILCOUNTRY ,  "Rwanda" )  OR  LIMIT-TO ( AFFILCOUNTRY ,  "Sudan" )  OR  LIMIT-TO ( AFFILCOUNTRY ,  "Zimbabwe" )  OR  LIMIT-TO ( AFFILCOUNTRY ,  "Botswana" )  OR  LIMIT-TO ( AFFILCOUNTRY ,  "Cameroon" )  OR  LIMIT-TO ( AFFILCOUNTRY ,  "Senegal" )  OR  LIMIT-TO ( AFFILCOUNTRY ,  "Tanzania" )  OR  LIMIT-TO ( AFFILCOUNTRY ,  "Zambia" ) OR  LIMIT-TO ( AFFILCOUNTRY ,  " Morocco")  OR  LIMIT-TO ( AFFILCOUNTRY,  "Angola")  OR  LIMIT-TO(AFFILCOUNTRY ,  "Mozambique")  OR  LIMIT-TO (AFFILCOUNTRY ,  "Madagascar" )  OR  LIMIT-TO ( AFFILCOUNTRY ,  " Côte d'Ivoire" ) OR  LIMIT-TO ( AFFILCOUNTRY ,  " Niger")  OR  LIMIT-TO (AFFILCOUNTRY ,  "Burkina Faso")  OR  LIMIT-TO(AFFILCOUNTRY ,  "Mali")  OR  LIMIT-TO (AFFILCOUNTRY ,  "Malawi" )  OR  LIMIT-TO ( AFFILCOUNTRY ,  " Chad" ) OR  LIMIT-TO ( AFFILCOUNTRY ,  " Somalia")  OR  LIMIT-TO (AFFILCOUNTRY ,  "Zimbabwe")  OR  LIMIT-TO(AFFILCOUNTRY ,  "Guinea")  OR  LIMIT-TO (AFFILCOUNTRY ,  "Malawi" )  OR  LIMIT-TO ( AFFILCOUNTRY ,  " Chad" )).

WOS

[192](tel:192) results from Web of Science Core Collection

Monocytogenes AND milk* (Topic) and Article (Document Type)
Refined By: Countries/Regions: Nigeria OR Ethiopia* OR Egypt* OR Congo* OR Tanzania* OR Africa* OR Kenya* OR Uganda* OR Algeria* OR Sudan* OR Morocco* OR Angola* OR Mozambique* OR Ghana* OR Madagascar* OR Cameroon* OR Côte d'Ivoire* OR Niger* OR Burkina* OR Mali* OR Malawi* OR Zambia* OR Senegal* OR Chad* OR Somalia* OR Zimbabwe* OR Guinea* OR Rwanda* OR Benin* OR Burundi* OR Tunisia* OR Togo* OR Sierra* OR Libya* OR Congo* OR Liberia* OR Mauritania* OR Eritrea* OR Namibia* OR Gambia* OR Botswana* OR Gabon* OR Lesotho* OR Guinea-Bissau* OR Equatorial?Guinea* OR Mauritius* OR Eswatini* OR Djibouti* OR Comoros* OR Cabo?Verde* OR Principe* OR Seychelles* OR Réunion * OR Western?Sahara* OR Mayotte* OR Helena*

Records identified from:

Databases (**N =405**):

- PubMed = **66**
- WOS = **192**
- Scopus = **145**

Records removed *before screening*:

Duplicate records removed (n =88)

**Identification**

**Included**

Reports assessed for eligibility

(n =57)

Reports excluded:

n=4

Studies included in systematic review (n =53) and disaggregated into 67 sub-studies in the models

Records screened

(**n =57)**

Records excluded

(n =**0**)

Reports sought for retrieval

(n =57)

Reports not retrieved

(n = 0)

**Screening**

**Identification of studies via databases**

**Figure S1. Schema for selecting studies on *Listeria monocytogenes* prevalence in milk in Africa.**

Results

Table S1. Detail sub-studies included in the **generalized logistic mixed-effects and meta-regression modelling of regional, subregional, and national prevalence of *Listeria monocytogenes* in milk in Africa.**

| Author | PY | P | N | Milk_type | Method | DNA_extraction | Nation | Subregion |
| --- | --- | --- | --- | --- | --- | --- | --- | --- |
| Ahmed et al. 2022 (Pasteurized) | 2022 | 0 | 60 | Pasteurized | CSP | Kit | Egypt | Northern Africa |
| Kayode and Okoh, 2022 (Pasteurized) | 2022 | 2 | 25 | Pasteurized | CSP | Boiling | South Africa | Southern Africa |
| Badawy et al. 2022 (Powdered) | 2022 | 9 | 160 | Powdered | CSP | Kit | Egypt | Northern Africa |
| Borena et al. 2022 (Raw) | 2022 | 9 | 384 | Raw | CS | NAS | Ethiopia | Eastern Africa |
| Ahimed et al. 2022 (Raw) | 2022 | 11 | 200 | Raw | CS | NAS | Ethiopia | Eastern Africa |
| Mogotu et al. 2022 (Raw) | 2022 | 5 | 92 | Raw | CS | NS | Kenya | Eastern Africa |
| Mohamed et al. 2022 (Raw) | 2022 | 8 | 150 | Raw | CSP | Kit | Egypt | Northern Africa |
| Elafify et al. 2022 (Raw) | 2022 | 6 | 50 | Raw | CSP | Kit | Egypt | Northern Africa |
| Ahmed et al. 2022 (Raw) | 2022 | 8 | 60 | Raw | CSP | Kit | Egypt | Northern Africa |
| Dapgh and Salem, 2022 (Raw) | 2022 | 6 | 25 | Raw | CSP | NS | Egypt | Northern Africa |
| Kayode and Okoh, 2022 (Raw) | 2022 | 7 | 26 | Raw | CSP | Boiling | South Africa | Southern Africa |
| Raufu et al. 2022 (Raw) | 2022 | 0 | 26 | Raw | CS | NAS | Nigeria | Western Africa |
| Ibrahim et al. 2021 (Powdered) | 2021 | 0 | 80 | Powdered | CP | NS | Egypt | Northern Africa |
| El Hag et al. 2021 (Raw) | 2021 | 3 | 720 | Raw | CS | NS | Sudan | Eastern Africa |
| Bouymajane et al. 2021 (Raw) | 2021 | 2 | 52 | Raw | CSP | Boiling | Morocco | Northern Africa |
| Abdeen et al. 2021 (Raw) | 2021 | 17 | 50 | Raw | CP | Kit | Egypt | Northern Africa |
| Rabehi et al. 2021 (Raw) | 2021 | 0 | 65 | Raw | CSP | Kit | Algeria | Northern Africa |
| Sarr et al. 2022 (Raw) | 2021 | 124 | 152 | Raw | CSP | Kit | Senegal | Western Africa |
| El-Gohary et al. 2020 (Raw) | 2020 | 8 | 120 | Raw | CS | NAS | Egypt | Northern Africa |
| Mohammed et al. 2020 (Raw/clinical mastitis cow's milk) | 2020 | 7 | 50 | Raw | CS | NAS | Egypt | Northern Africa |
| Togo et al. 2020 (Raw) | 2020 | 9 | 10 | Raw | CP | NS | Mali | Western Africa |
| El-Demerdash and Raslan, 2019 (Raw) | 2019 | 1 | 50 | Raw | CP | Kit | Egypt | Northern Africa |
| Bouymajane et al. 2019 (Raw) | 2019 | 7 | 36 | Raw | CS | NAS | Morocco | Northern Africa |
| Owusu-Kwarteng et al. 2018 (Boiled) | 2018 | 10 | 56 | Boiled | CSP | Boiling | Ghana | Western Africa |
| Owusu-Kwarteng et al. 2018 (Nunu/Fermented) | 2018 | 11 | 84 | Fermented | CSP | Boiling | Ghana | Western Africa |
| Owusu-Kwarteng et al. 2018 (Raw) | 2018 | 14 | 144 | Raw | CSP | Boiling | Ghana | Western Africa |
| Tahoun et al. 2017 (Raw) | 2017 | 69 | 300 | Raw | CP | Kit | Egypt | Northern Africa |
| Nzabuheraheza and Nyiramugwera, 2016 (Fermented) | 2016 | 0 | 4 | Fermented | CS | NAS | Rwanda | Eastern Africa |
| Osman et al. 2016 (Raw) | 2016 | 3 | 203 | Raw | CSP | Boiling | Egypt | Northern Africa |
| Reda et al. 2016 (Raw) | 2016 | 4 | 50 | Raw | CP | Kit | Egypt | Northern Africa |
| Barkallah et al. 2016 (Raw) | 2016 | 10 | 53 | Raw | CP | Kit | Tunisia | Northern Africa |
| Usman et al. 2016 (Raw) | 2016 | 9 | 36 | Raw | CP | Kit | Nigeria | Western Africa |
| Seyoum et al. 2015 (Pasteurized) | 2015 | 13 | 65 | Pasteurized | CS | NAS | Ethiopia | Eastern Africa |
| Garedew et al. 2015 (Raw) | 2015 | 2 | 50 | Raw | ACP | NAS | Ethiopia | Eastern Africa |
| Seyoum et al. 2015 (Raw) | 2015 | 7 | 343 | Raw | CS | NAS | Ethiopia | Eastern Africa |
| Farouk et al. 2015 (Raw) | 2015 | 2 | 60 | Raw | ACP | Kit | Egypt | Northern Africa |
| Boubendir et al. 2015 (Raw) | 2015 | 0 | 104 | Raw | CP | Kit | Algeria | Northern Africa |
| Belbachir et al. 2015 (Raw) | 2015 | 2 | 80 | Raw | CS | NAS | Morocco | Northern Africa |
| Kamana et al. 2014 (boiled) | 2014 | 0 | 42 | Boiled | CS | NAS | Rwanda | Eastern Africa |
| Kamana et al. 2014 (Fermented) | 2014 | 0 | 6 | Fermented | CS | NAS | Rwanda | Eastern Africa |
| Kamana et al. 2014 (Pasteurized) | 2014 | 0 | 48 | Pasteurized | CS | NAS | Rwanda | Eastern Africa |
| Kamana et al. 2014 (Raw) | 2014 | 0 | 174 | Raw | CS | NAS | Rwanda | Eastern Africa |
| Osman et al. 2014a (Raw) | 2014 | 60 | 209 | Raw | CSP | Boiling | Egypt | Northern Africa |
| Hmaied et al. 2014 (Raw) | 2014 | 2 | 20 | Raw | CSP | Boiling | Tunisia | Northern Africa |
| Al-Ashmawy et al. 2014 (Raw/bulk tank) | 2014 | 16 | 100 | Raw | CSP | Boiling | Egypt | Northern Africa |
| Ismaiel et al. 2014 (Raw) | 2014 | 0 | 30 | Raw | CS | NAS | Egypt | Northern Africa |
| Osman et al. 2014b (Raw) | 2014 | 1 | 100 | Raw | CSP | NS | Egypt | Northern Africa |
| Schoder et al. 2013 (Fermented) | 2013 | 0 | 46 | Fermented | CS | NAS | Tanzania | Eastern Africa |
| Schoder et al. 2013 (Pasteurized) | 2013 | 0 | 41 | Pasteurized | CS | NAS | Tanzania | Eastern Africa |
| Derra et al. 2013 (Raw) | 2013 | 5 | 60 | Raw | CSP | Boiling | Ethiopia | Eastern Africa |
| Schoder et al. 2013 (Raw) | 2013 | 0 | 109 | Raw | CS | NAS | Tanzania | Eastern Africa |
| El-Gama et al. 2013 (Raw) | 2013 | 12 | 70 | Raw | CS | NAS | Egypt | Northern Africa |
| Alall et al. 2012 (Raw) | 2012 | 3 | 185 | Raw | CSP | Boiling | Egypt | Northern Africa |
| Hadrya et al. 2012 (Raw) | 2012 | 1 | 120 | Raw | CS | NAS | Morocco | Northern Africa |
| Bouazza et al. 2012 (Raw) | 2012 | 0 | 30 | Raw | CS | NAS | Morocco | Northern Africa |
| Yakubu et al. 2012 (Raw) | 2012 | 11 | 288 | Raw | CSP | NS | Nigeria | Western Africa |
| Gebretsadik et al. 2011 (Raw) | 2011 | 22 | 100 | Raw | CS | NAS | Ethiopia | Eastern Africa |
| Boubendir et al. 2011 (Raw) | 2011 | 1 | 104 | Raw | CSP | Boiling | Algeria | Northern Africa |
| Mengesha et al. 2009 (Pasteurized) | 2009 | 0 | 50 | Pasteurized | CS | NAS | Ethiopia | Eastern Africa |
| Morobe et al. 2009 (Raw) | 2009 | 3 | 300 | Raw | CS | NAS | Botswana | Southern Africa |
| Hamdi et al. 2007 (Pasteurized) | 2007 | 6 | 80 | Pasteurized | CSP | NS | Algeria | Northern Africa |
| Hamdi et al. 2007 (Raw) | 2007 | 4 | 153 | Raw | CSP | NS | Algeria | Northern Africa |
| Arimi et al. 1997 (Pasteurized) | 1997 | 5 | 26 | Pasteurized | CS | NS | Kenya | Eastern Africa |
| Arimi et al. 1997 (Raw) | 1997 | 3 | 14 | Raw | CS | NAS | Kenya | Eastern Africa |
| Marrakchi et al. 1993 (Fermented) | 1993 | 5 | 63 | Fermented | CS | NS | Morocco | Northern Africa |
| Marrakchi et al. 1993 (Pasteurized) | 1993 | 0 | 20 | Pasteurized | CS | NAS | Morocco | Northern Africa |
| Marrakchi et al. 1993 (Raw) | 1993 | 3 | 30 | Raw | CS | NAS | Morocco | Northern Africa |

**Table S2: Descriptive summary of included/disaggregated studies.**

| Characteristic | N = 67*^1^* |
| --- | --- |
| P (positive) | 8.48 ±18.28 |
| Skewness ±SE | 4.83±0.29 |
| Kurtosis ±SE | 26.39±0.58 |
| N (sample size) | 102.88 ±113.12 |
| Skewness ±SE | 3.09±0.29 |
| Kurtosis ±SE | 13.08±0.58 |
| Milk type |  |
| Boiled milk | 2/67 (3.0%) |
| Fermented milk | 5/67 (7.5%) |
| Pasteurized milk | 9/67 (13%) |
| Powdered milk | 2/67 (3.0%) |
| Raw milk | 49/67 (73%) |
| *L. monocytogenes* detection method |  |
| ACP (API kit, cultural and PCR) | 2/67 (3.0%) |
| CP (cultural and PCR) | 9/67 (13%) |
| CS (cultural and serology) | 31/67 (46%) |
| CSP (cultural, serology, and PCR) | 25/67 (37%) |
| DNA extraction approach |  |
| Boiling | 13/67 (19%) |
| Kit | 15/67 (22%) |
| Not applicable | 28/67 (42%) |
| Not specified | 11/67 (16%) |
| Nation |  |
| Algeria | 5/67 (7.5%) |
| Botswana | 1/67 (1.5%) |
| Egypt | 21/67 (31%) |
| Ethiopia | 8/67 (12%) |
| Ghana | 3/67 (4.5%) |
| Kenya | 3/67 (4.5%) |
| Mali | 1/67 (1.5%) |
| Morocco | 8/67 (12%) |
| Nigeria | 3/67 (4.5%) |
| Rwanda | 5/67 (7.5%) |
| Senegal | 1/67 (1.5%) |
| South Africa | 2/67 (3.0%) |
| Sudan | 1/67 (1.5%) |
| Tanzania | 3/67 (4.5%) |
| Tunisia | 2/67 (3.0%) |
| Subregion |  |
| Eastern Africa | 20/67 (30%) |
| Northern Africa | 36/67 (54%) |
| Southern Africa | 3/67 (4.5%) |
| Western Africa | 8/67 (12%) |
| *^1^*Mean ±SD; n/N (%) | |
|  | |

**Table S3: Subregional specific distribution and description of studies on *L. monocytogenes* contamination of milk in Africa.**

| Characteristic | Eastern Africa, N = 20*^1^* | Northern Africa, N = 36*^1^* | Southern Africa, N = 3*^1^* | Western Africa, N = 8*^1^* |
| --- | --- | --- | --- | --- |
| P | 4.25±5.80 | 7.86 ±14.65 | 4.00 ±2.65 | 23.50 ±40.81 |
| N | 128.70 ±173.88 | 88.11 ±62.81 | 117.00 ±158.48 | 99.50 ±92.46 |
| Nation |  |  |  |  |
| Algeria |  | 5/36 (14%) |  |  |
| Botswana |  |  | 1/3 (33%) |  |
| Egypt |  | 21/36 (58%) |  |  |
| Ethiopia | 8/20 (40%) |  |  |  |
| Ghana |  |  |  | 3/8 (38%) |
| Kenya | 3/20 (15%) |  |  |  |
| Mali |  |  |  | 1/8 (12%) |
| Morocco |  | 8/36 (22%) |  |  |
| Nigeria |  |  |  | 3/8 (38%) |
| Rwanda | 5/20 (25%) |  |  |  |
| Senegal |  |  |  | 1/8 (12%) |
| South Africa |  |  | 2/3 (67%) |  |
| Sudan | 1/20 (5.0%) |  |  |  |
| Tanzania | 3/20 (15%) |  |  |  |
| Tunisia |  | 2/36 (5.6%) |  |  |
| *^1^*Mean ±SD; n/N (%) | | | | |
